# Surgical Management of Acquired Strabismus Resulting From Ocular Trauma

**DOI:** 10.7759/cureus.39047

**Published:** 2023-05-15

**Authors:** Rasika Bagewadi, Sachin Daigavane

**Affiliations:** 1 Department of Ophthalmology, Jawaharlal Nehru Medical College, Datta Meghe Institute of Medical Sciences, Sawangi (Meghe), Wardha, IND

**Keywords:** paralytic strabismus, ocular injury, ocular trauma, acquired strabismus, strabismus

## Abstract

Strabismus is a disorder in which the eyes are incorrectly lined up with each other. Either eye is always or infrequently looking inward (esotropia) or outward (exotropia). A 19-year-old male patient came to the Ophthalmology Outpatient Department (OPD) with complaints of outward deviation of the left eye for five years. It was associated with a diminution of vision in the left eye for three years. The patient reported a history of a road traffic accident (RTA) five years ago before the development of deviation of the left eye. On examination, the Hirschberg test showed a corneal light reflex falling beyond the limbus. After obtaining due consent for anesthesia risk and medicine fitness, the patient underwent squint correction surgery (medial rectus resection) and was started on oral and topical antibiotics with a 15-day follow-up period. Postoperative orthophoria was achieved.

## Introduction

Strabismus is a disorder in which the eyes are incorrectly lined up with each other. Either eye is always or infrequently looking inward (esotropia) or outward (exotropia) [1]. Strabismus resulting from orbital or ocular trauma can be due to local factors like swelling or due to orbital fracture, complete or partial loss of extraocular muscle (EOM), loss of function of cranial nerve, or damage to structures in the surrounding area. This type of strabismus is usually incomitant and can be very difficult to manage. It can result in diplopia, which can hamper a person’s everyday activities. If there is an impairment in visual function due to the ocular injury, it can lead to secondary strabismus [2]. The patient may present with a wide range of symptoms, which can vary from mild to severely hampering the patient’s well-being. Life-threatening trauma may also be associated with ocular injury. Hence, along with the management of the ocular injury, the patient’s overall condition also needs to be looked after. This case report presents one such case of strabismus after a car accident and its management.

## Case presentation

A 19-year-old male patient came to the Ophthalmology Outpatient Department (OPD) with primary complaints of outward deviation of the left eye for five years, along with diminution of vision in the left eye for three years. The patient reported a history of a road traffic accident five years ago before the development of deviation of the left eye. On clinical examination and initial evaluation, vitals were stable, and he was afebrile. A general examination was performed, and no abnormalities were detected. His BMI was normal. His blood pressure was 130/80 mmHg. On neurological evaluation, he was conscious and oriented. Findings for meningeal irritation or any cranial nerve abnormalities were not found.

**Figure 1:**
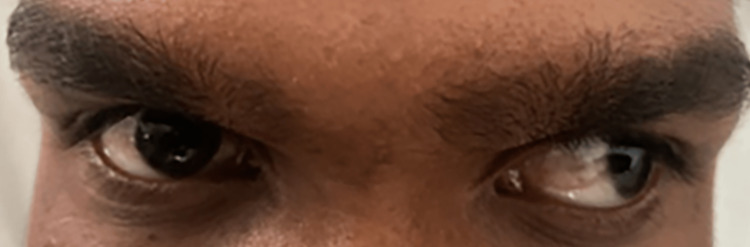
Exotropia of 60° in the left eye.

Ophthalmologic examination revealed the best-corrected visual acuity with -2 diopter sphere (DS) in the right eye was 6/9 and with -1.75 DS in the left eye was 6/9. Color vision in both eyes was tested with the 14 plate Ishihara test, which had a result of 14/14, and a visual field examination using automated perimetry found no visual field defects. Hirschberg test in the right eye showed a corneal light reflex falling centrally at the pupil. Extraocular movement examination showed impaired adduction in the left eye. In the left eye, the Hirschberg test showed a corneal light reflex falling beyond the limbus with a deviation of 60°. The cover-uncover test revealed exotropia of 60° in the left eye (Figure 1). The primary and secondary deviation was almost equal of 60 prism diopter (PD).

The Worth’s Four dot test was suggestive of no suppression, but diplopia was seen. The prism bar cover test showed exotropia of 60 PD in the left eye. The posterior segment evaluation of both eyes was within normal limits. Extraocular movements revealed reduced adduction in the left eye.

**Figure 2:**
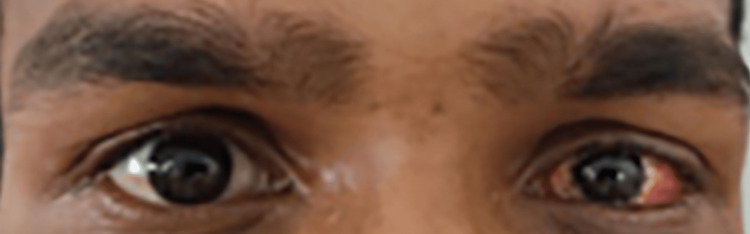
Postoperative orthophoria was achieved.

On radiological investigation, X-ray orbit was done, which was found to be within normal limits. CT scan revealed no bony abnormality or muscle entrapment. On therapeutic intervention, based on clinical findings, the patient was most probably a case of paralytic strabismus of the left eye and was taken up for squint correction surgery. After obtaining due consent for general anesthesia risk and medicine fitness, squint correction surgery (medial rectus resection) was done. A lid speculum was placed. The forced duction test was negative. A limbal incision was made. Dissection was performed to the bare sclera through the conjunctiva, Tenon's capsule, and intermuscular septum. A muscle hook was then passed between Tenon's capsule and sclera. The fascial attachments and ligaments of the muscle were cleaned. For resection, calipers were used to measure the amount of muscle to be resected from the insertion. A suture was then used to secure the muscle. The muscle was disinserted from the globe, and the stump of insertion was held. A 6 mm portion of the medical rectus muscle to be resected was excised from the clamp. The cut muscle edge was then cauterized. The sutures were tied and cut. The conjunctiva was approximated and closed. The patient was started on oral and topical antibiotics with a 15-day follow-up period. Postoperative orthophoria was achieved (Figure 2).

## Discussion

Strabismus is an entity wherein both eyes are abnormally lined up with each other. This may be accompanied by deranged movement of one or both eyes, diplopia, reduced visual acuity, headache, or abnormal head position and posture [1]. Strabismus, which occurs due to ocular or orbital trauma, can result from localized factors or following orbital fractures, partial or complete loss of EOM or cranial nerve function, or damage to surrounding tissues, which results in mechanical restriction [2]. The strabismus caused by such causes is usually incomitant, and it can be challenging to entirely correct. The surgery may result in diplopia, affecting the person’s daily life [3].

Multifactorial etiologies may be causative for strabismus, which can be related to an ocular cause, orbital cause, or EOM trauma. All of these need individual and specific treatment. Localized acute development like local edema, hemorrhage, and soft tissue swelling may lead to diplopia and visual problems. Orbital fractures causing muscle entrapment, any cranial nerve palsies, EOM disinsertion, or direct trauma to the EOM or adjacent tissue can lead to a chronic sequel, and these need management by a skilled surgeon or a strabismologist. Any pre-existing conditions causing sensory deficit may be a confounding factor in a neurological examination. Therefore, a thorough neurological examination is critical in such cases [4]. 

There can be a decrease in visual acuity after an ocular injury that can result in secondary strabismus. The patient may present with a wide range of symptoms, which can vary from mild to severely hampering the patient’s well-being. Life-threatening trauma may also be associated with ocular injury. Hence, along with the management of the ocular injury, the patient’s overall condition also needs to be looked after [1].

## Conclusions

EOM trauma without globe or eyelid involvement rarely occurs but can be seen. A thorough evaluation of the globe should be mandatory to check for any traumatic injury to the globe. Assessing the condition of the affected muscle is of utmost significance for planning the most appropriate management of the case. Surgical correction offers good cosmetic results. This case report presents one such case of medial rectus palsy in a young male following a road traffic accident and emphasizes the need for thorough evaluation and further management.
